# Swine farm environmental microbiome: exploring microbial ecology and functionality across farms with high and low sanitary status

**DOI:** 10.3389/fvets.2024.1401561

**Published:** 2024-07-03

**Authors:** Clara Marin, Lourdes Migura-García, Juan Carlos Rodríguez, María-Paz Ventero, Maria Teresa Pérez-Gracia, Santiago Vega, Carla Tort-Miró, Ana Marco-Fuertes, Laura Lorenzo-Rebenaque, Laura Montoro-Dasi

**Affiliations:** ^1^Facultad de Veterinaria, Instituto de Ciencias Biomédicas, Universidad Cardenal Herrera-CEU, CEU Universities, Valencia, Spain; ^2^IRTA, Programa de Sanitat Animal, CReSA, Collaborating Centre of the World Organisation for Animal Health for Research and Control of Emerging and Re-Emerging Pig Diseases in Europe, Barcelona, Spain; ^3^Unitat mixta d’Investigació IRTA-UAB en Sanitat Animal, Centre de Recerca en Sanitat Animal (CReSA), Campus de la Universitat Autònoma de Barcelona, Barcelona, Spain; ^4^Microbiology Department, Dr. Balmis University General Hospital, Microbiology Division, Miguel Hernández University, ISABIAL, Alicante, Spain; ^5^Área de Microbiología, Departamento de Farmacia, Instituto de Ciencias Biomédicas, Facultad de Ciencias de la Salud, Universidad Cardenal Herrera-CEU, CEU Universities, Valencia, Spain; ^6^Institute of Science and Animal Technology, Universitat Politècnica de Valencia, Valencia, Spain

**Keywords:** environmental microbiome, pig production, 16S rRNA sequencing, farm health status, cleaning and disinfection protocols

## Abstract

**Introduction:**

Stringent regulations in pig farming, such as antibiotic control and the ban on certain additives and disinfectants, complicate disease control efforts. Despite the evolution of microbial communities inside the house environment, they maintain stability over the years, exhibiting characteristics specific to each type of production and, in some cases, unique to a particular company or farm production type. In addition, some infectious diseases are recurrent in specific farms, while other farms never present these diseases, suggesting a connection between the presence of these microorganisms in animals or their environment. Therefore, the aim of this study was to characterise environmental microbiomes of farms with high and low sanitary status, establishing the relationships between both, health status, environmental microbial ecology and its functionality.

**Methods:**

For this purpose, 6 pig farms were environmentally sampled. Farms were affiliated with a production company that handle the majority of the pigs slaughtered in Spain. This study investigated the relationship among high health and low health status farms using high throughput 16S rRNA gene sequencing. In addition, to identify ecologically relevant functions and potential pathogens based on the 16S rRNA gene sequences obtained, functional Annotation with PROkaryotic TAXa (FAPROTAX) was performed.

**Results and Discussion:**

This study reveals notable differences in microbial communities between farms with persistent health issues and those with good health outcomes, suggesting a need for protocols tailored to address specific challenges. The variation in microbial populations among farms underscores the need for specific and eco-friendly cleaning and disinfection protocols. These measures are key to enhancing the sustainability of livestock farming, ensuring safer products and boosting competitive edge in the market.

## 1 Introduction

Decisions made by European authorities, such as the prohibition of antibiotics as prophylactics (since 2006), the recently restricted use of colistin and the banning of zinc oxide as treatment, have led to a significant increase in on-farm diseases, such as *Salmonella* spp. or post-weaning diarrhoea (1). Moreover, this leads to an increase in the excretion of these pathogens into the environment and, consequently, an increase in environmental infectious pressure and alteration of the microbial ecology of the farm environment (2). For all these reasons, searching for cost-effective, animal-friendly, and environmentally respectful alternatives to antibiotics are necessary; all of them aim to enable the animal to be immunocompetent, resilient to fight infectious diseases and to recover as quickly as possible in the event of illness (3).

Among the various alternatives to antibiotics at field level, the most studied are management, the development of new vaccines, feed supplements, and strict biosecurity, including comprehensive cleaning and disinfection (C&D) protocols. Martelli et al. (4) demonstrated that an adequate C&D schedule significantly reduces residual contamination in pig facilities. However, persistent pathogens such as *Salmonella* spp. remain in the environment among batches. Therefore, routine C&D protocols used in the field must be complemented with other control measures throughout the pig breeding and production chain (4).

In recent years, an increasing number of studies have highlighted the potential role of biocides in selecting cross-resistance to antibiotics in bacteria. However, the vast majority of them were conducted using pure culture models under laboratory conditions (5, 6). The current absence of field data makes it difficult to assess the significant risk of selecting bacteria with heightened antibiotic resistance due to the use of biocides in industries, as well as to determine which biocides are linked to the highest risk of cross-selection of antibiotic resistance (7).

Traditionally, different types of disinfectants have been used at field level for surface hygiene in livestock, such as products based on quaternary ammonium compounds containing glutaraldehyde, formaldehyde, peroxide-based compounds or peracetic acid, iodine-based compounds or chlorocresols. However, it is widely known that formaldehyde is one of the most effective disinfectants against zoonotic pathogens of such importance as *Salmonella* spp. (8). Restrictions in Europe on the use of formaldehyde, given its categorisation as a carcinogen, mutagen, and highly toxic, have meant the imminent need for the sector to seek alternatives for farm hygiene (8, 9). In this context, C&D is key to reducing the environmental pressure of pathogens and the number of infected individuals, but the threat of persistence of the most resistant strains in the environment remains, as they can persist between consecutive animal batches, increasing the risk of infection (10).

Farm environment can be a complex stable community of microorganisms, mostly bacteria, but also includes viruses, archaea, fungi, yeasts and protozoa, that persists among different animal batches. The microorganisms that inhabit the farm environment can strongly influence the associated animal health, food quality and safety (11). In this sense, there is a growing research effort to unravel the ecological mechanisms that encompass this microbiome. Among them, it is noteworthy that these communities may find protection through structures known as biofilms. Biofilms are formations attached to a surface where microorganisms are embedded and shielded by an exopolysaccharide matrix. The forming of biofilms improves the producer’s ability to endure in a specific environment (12, 13). Moreover, communities safeguarded by a biofilm exhibit 100 times more resistance against C&D protocols (14, 15). Additionally, adaptive stress responses contribute to the efficiency of these communities in surviving the farm environment (16).

Despite the evolution of microbial communities inside the house environment (11), these communities maintain stability over the years, exhibiting characteristics specific to each type of production and, in some cases, unique to a particular company or production type (17). On several occasions, clones of microorganisms, such as *Salmonella* spp., or *Campylobacter* spp., found in animal-derived products have been commonly detected at the farm of origin (18, 19). In addition, some infectious diseases are always associated with specific farms, while other farms never present diseases (high health status farms *vs* low health status farms), suggesting a connection between the presence of these microorganisms in the animals and their surrounding environment at the farm.

Thus, the aim of this study was to characterise environmental microbiomes of both, high and low sanitary status farms, establishing the relationships between health status, environmental microbial ecology and its functionality.

## 2 Materials and methods

### 2.1 Study sample

Over a 4-month period, 6 houses from 6 pig farms were environmentally sampled. Farms were affiliated with a production company that handles the majority of the pigs slaughtered in Spain.

### 2.2 Selection of high and low sanitary status farms to define “healthy” and “unhealthy” microbiomes

A farm with a High Health Status (HHS) should meet the following criteria: (i) An outstanding production performance. Production parameters measured as average daily gain and feed conversion rate falling within the best 25% of the whole pig population. (ii) Outstanding health records reaching at least the best 10% of the whole pig population. These parameters are mortality, percentage of substandard pigs during the rearing period and treatment cost due to antimicrobials. The expected mortality rate from weaning to slaughter, percentage of substandard pigs and treatment cost for these farms would be 4%, 2–3% and 1–1.5 € per pig, respectively. These farms are usually selection and multiplication farms.

Accordingly, Low Health Status (LHS) farms in this case will be those farms with recurrent problems of post-weaning diarrhoea within 2 weeks after weaning and at least 10–15% morbidity.

Based on the information provided by the company enrolled for the study, management practices were standardised across all their farms. Still, HHS and LHS farms have all passed the Biocheck UGent survey. However, farms with HHS scored higher for some of the features included in both, external and internal biosecurity, especially, “purchase of breeding pigs,” “personnel and people visiting the farm,” “measures between compartments, “working lines and use of equipment” and “cleaning and disinfection.” Along with the Biocheck UGent, the company had established its own risk index for the main swine pathogens based on their occurrence at their farms during the last 10 years. HHS farms were free of these main swine pathogens; porcine reproductive and respiratory syndrome virus (PRRSV), swine dysentery and *Mycoplasma hyopneumoniae* and had mortality rates lower than 4%.

### 2.3 Sampling

**Figure 1:**
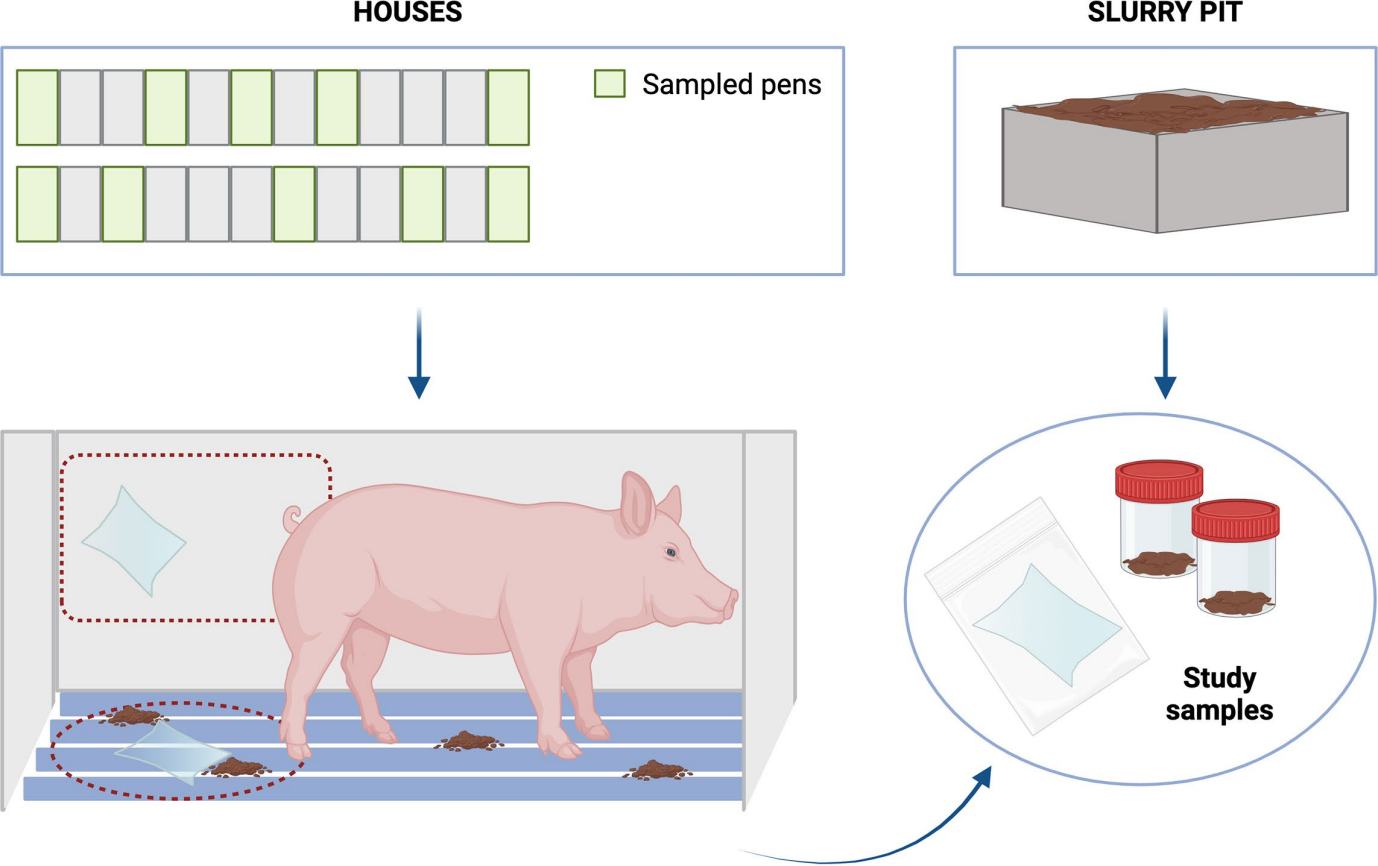
Sampling scheme.

During January and June 2023, six pig farms were enrolled for the study (3 HHS and 3 LHS). One house from each farm was selected according to the described criteria. At each farm, one house from the nursery facility containing sows with 3 weeks-old piglets was selected. Thereafter, 10 pens within each house were sampled (walls and slats), i.e., four and six pens from the corners and the middle of the house, respectively (Figure 1). In addition, two slurry pit samples were collected. For this purpose, two 500 mL sterile pots were filled from different points of the house pits to ensure a representative sample from each house studied. Wall and slat samples were collected by wiping 1 m^2^ of surfaces using both sides of the sterile wipe (Whirl-Pak^®^, Scharlab, Madrid). First, wall samples (10 per farm, one per pen) were taken at an approximate height of 70 cm above the pen floor. Then, the slat samples (10 per farm, one per pen) were collected, between and under the grates whenever possible. Once collected, all samples were placed individually in a sterile bag with sterile diluent and transported to the laboratory in refrigerated conditions (4°C) and processed within 12 h.

Once in the laboratory, wall and slat samples were processed identically. Two pools of 5 wipes each (1/4 of a wipe per pool) were generated for each type of sample. The remaining wipes were stored in freezer for further studies. Then, each pool and each slurry sample were placed in a stomacher (BagFilter^®^ 400 mL, Scharlab, Madrid) and homogenised with 15 mL of PBS (5 min, 260 rpm).

### 2.4 DNA extraction, 16S rRNA gene amplification, and MiSeq sequencing

The DNA was extracted from 250 μL of each homogenised environmental sample (wall and slats) and from 250 μL of slurry following the manufacturer’s instructions (QIAamp Power Fecal Pro DNA kit, Werfen, Barcelona, Spain). The DNA quality was determined using Nanodrop ND − 1000 spectrophotometer (Thermo Scientific, Wilmington, DE, United States) and DNA was quantified using Qubit fluorometer (Life Technologies, Paisley, United Kingdom). The DNA was frozen at −20°C for shipment at the *Instituto de Investigación Sanitaria y Biomédica de Alicante*—ISABIAL (Alicante, Spain). Once there, 16S rRNA gene amplification and MiSeq (Illumina) sequencing was performed according to Montoro-Dasi et al. (20). To this end, 12.5 ng of DNA from each sample, quantified using Qubit, was used to prepare the library according to the 16S rRNA Metagenomic Sequencing Library Preparation protocol (Illumina). The primers targeted the V3 and V4 regions of the 16S rRNA gene, and included Illumina adapters: 16S Amplicon PCR Forward Primer = 5′ TCGTCGGCAGCGTCAGATGTGTATAAGAGACAGCCTACGGGNGGCWGCAG; and 16S Amplicon PCR Reverse Primer = 5′ GTCTCGTGGGCTCGGAGATGTGTATAAG AGACAGGA CTACHVGGGTATCTAATCC. NGS Libraries were analysed using the Agilent 4200 TapeStation System to ensure their integrity. Sequencing was performed on a MiSeq (21) system in 2 × 300 bp format. The quality of the raw reads was evaluated using the FastQC software (22).

### 2.5 Bioinformatic analysis

Bioinformatic analyses were performed separately for each type of sample (wall, slat, and slurry). For this purpose, the Amplicon Sequence Variant (ASV) picking, and analysis was performed with QIIME2 (v2021.4) pipeline (23). Demultiplexed paired FASTQ sequences were imported into the QIIME2 v2021.4. The DADA2 plugin incorporated into QIIME2 was used to quality filter, denoise, combine, and remove chimaera from the sequences. The raw sequences were truncated from the first low-quality base site whose number of low-quality values (default quality threshold ≤30) and the primer sequences were removed from all reads. Then, sequences were grouped into amplicon sequence variants (ASVs) with 99% identification. Taxonomy was assigned to ASVs using the classify-sklearn naïve Bayes taxonomy classifier in the feature-classifier plugin against the SILVA v138 database (24, 25). Sequences not assigned to any taxa or classified as *Eukaryote*, *Archaea* or only bacteria were filtered out (24, 25).

### 2.6 Statistical analysis

The statistical analysis of wall, slats and slurry was conducted using the following methodology (26). No outlier samples were identified via principal component analysis. Genera with almost 25% zeros within each treatment were excluded. The remaining zeros were replaced by one for microbiome data and by half of the minimum value detected for each genus. A total of 57, 95, and 259 genera, from the wall, slat and slurry samples, respectively, remained in the datasets. Datasets were transformed using the additive log-ratio (ALR) transformation as follows (Equation 1):


(1) $$AL\left(j||ref\right)=\log\left(\mathrm{xjxref}\right)=\log\left(xj\right)-\log\left(\mathrm{xref}\right)$$

Here, j represents the total number of variables in the dataset, 𝑥j is the value for the genus j and 𝑥𝑟𝑒𝑓 is the reference variable used for data transformation. The reference variable was chosen as the one with the lowest coefficient of variation: Cellulosilyticum for wall samples, p-2534-18B5_gut_group for slat samples, and Dojkabacteria for slurry samples. The lack of isometry was checked using Procrustes correlation (27). ALRs were autoscaled with mean of 0 and standard deviation of 1.

A partial least-squares discriminant analysis (PLS-DA) was employed to identify the genera that allowed the classification or discrimination of the sanitary status (28). For the PLS-DA models, the sanitary status served as the categorical vector y, and the ALR-transformed dataset for genera was utilised as the matrix X. The balance error rate (BER) for the Mahalanobis distance, calculated using a 4-fold cross-validation repeated 100 times, was employed to determine the optimal number of components for the model in each iteration. During each iteration, variables with a variable importance prediction (VIP) score below 1 were excluded from the X matrix, as they did not contribute significantly to the classification among the treatments (29). Following variable selection, a new PLS-DA model was computed. This process of variable selection and PLS-DA model computation were repeated until the lowest BER was obtained. The prediction accuracy of the final PLS-DA model was assessed by constructing a confusion matrix and a permuted confusion matrix, using 4-fold cross-validation repeated 10,000 times. The confusion matrix enabled the evaluation of the model’s ability to predict each treatment based on the selected variables, while the permuted confusion matrix assessed whether the observed performance was due to a random selection of variables throughout the PLS-DA iterations. The prediction performance was deemed spurious when the percentage of true positives for each treatment was far from their random probabilities (50% for two categories).

In addition to PLS-DA, Bayesian statistics were employed to assess the relevance of the differences in genera abundance between the HHS and LHS groups. A model with a single effect of “environment” and flat priors was fitted. The marginal posterior distribution of the unknowns was estimated using MCMC with four chains of 50,000 iterations, with a burn-in of 1,000 and a lag of 10. The posterior mean of the differences between the HHS and LHS was used to estimate the posterior mean of the differences in genera abundance between these groups. These estimates were expressed in units of standard deviation (SD) for each variable. Differences in the mean abundance of genera between the HHS and the LHS groups were considered relevant when the probability of the differences (30) being greater (if the difference is positive) or lower (if negative) than 0 (P0) was higher than 0.95.

Alpha and beta diversity were calculated using the ALR at the genus level to assess differences in microbiome composition among groups in wall, slat and slurry samples. Alpha diversity was evaluated with the Shannon diversity and the inverse Simpson indexes to analyse the species diversity and evenness. Differences in alpha diversity distribution among groups were considered significant when the *p* value from a Mann–Whitney U test was lower than 0.05. Beta diversity was assessed using the Bray–Curtis dissimilarity matrix, and a nonmetric multidimensional scaling (NMDS) was performed to obtain the loadings of the first two dimensions. Differences in microbial genera composition were examined using a permutational multivariate analysis of variance (PERMANOVA; *p* value < 0.05) on the loadings of the two first MDS dimensions (31, 32).

### 2.7 Bacterial functional annotation and distribution

Finally, the Functional Annotation of PROkaryotic TAXa (FAPROTAX) database (33, 34) was implemented to identify ecologically relevant functions, potential pathogens based on the 16S rRNA gene sequences for each sample, and for more-related-environmental samples (wall and slat).

## 3 Results

A total of 36 samples were collected from 3 HHS and 3 LHS farms. On each farm, 10 samples from wall (2 pooled samples/farm), 10 from slat (2 pooled samples/farm), and 2 from slurry pit were collected.

### 3.1 16S rRNA sequencing

The summary of sequences and ASVs obtained in this study for wall, slat, and slurry samples can be seen in Table 1, detailing the results obtained from each sample type.

**Table 1:** General features of 16S rRNA amplicon sequencing of wall, slat, and slurry microbiota.

	Wall	Slat	Slurry
Total raw reads	1,256,874	1,184,076	1,365,695
Average sequence length (bp)	417.8	416.6	411.8
Average number of sequences for sample	104,739.5	98,676	113,807.9
Total sequences	744,382	724,024	812,647
ASV’s generated	1,354	1,926	5,892
ASV’s generated for taxonomic assignment	284	376	1,268

bp, base pairs; ASVs, amplicon sequence variants.

The datasets generated and analysed are available at NCBI’s BioProject PRJNA1079579 and BioSample SAMN40044702.

### 3.2 Taxonomic characterisation of environmental microbial communities

To better establish the microbial community composition of pig farm environments according to their health status, organisms present at different taxonomic levels and their relative abundance were evaluated. Alignment of ASVs against the SILVA database resulted in identification of 23 bacterial *phyla* and 389 bacterial genera. While the majority of OTUs were identified at genus level (255), some were only classified at the *phylum*, class, order, or family level.

**Figure 2:**
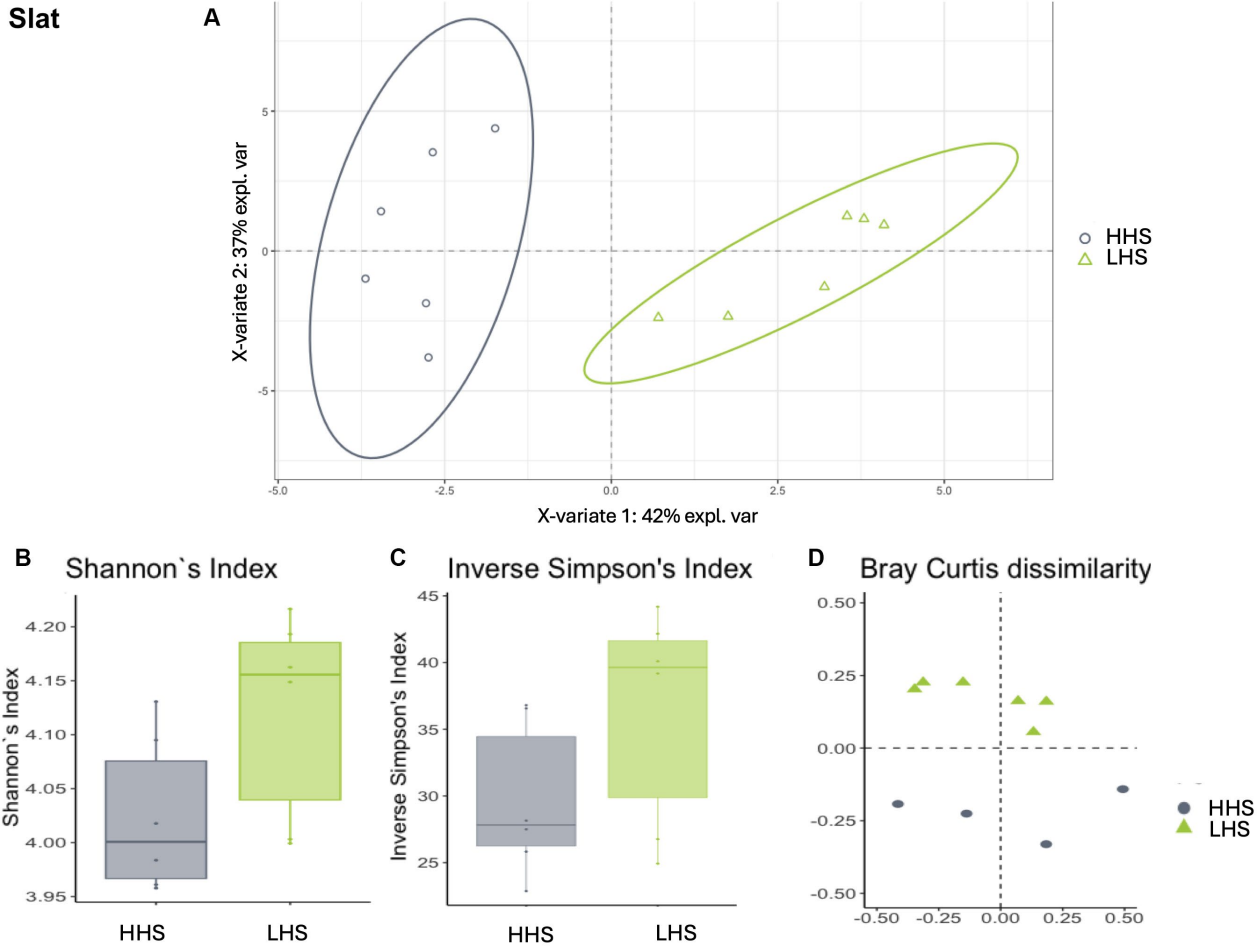
Environmental microbiota modulation induced by sanitary status in pig farms in wall samples. **(A)** Two-dimensional PLS-DA score plot was constructed using microbiota genera as variables, representing the distribution of the samples between the first two components in the model, which correspond to the sanitary status (HHS vs. LHS). Alpha diversity was computed using the **(B)** Shannon diversity index and **(C)** inverse Simpson index. Beta diversity was computed by calculating **(D)** the Bray–Curtis dissimilarity matrix. Differences among populations were established as having a *p*-value lower than 0.05. HHS, high health status; LHS, low health status.

At *phylum* level, *Firmicutes* represented the dominant phylum of the environmental community in all the sample types collected. In the case of wall and slat samples, this *phylum* was followed by *Proteobacteria*. For wall samples, relative abundance of *Proteobacteria* comprised 25.0 and 25.4%, for HHS and LHS farms, respectively, of total relative abundance, whereas for slat samples was 21.3 and 18.5%, respectively. For slurry samples, *Firmicutes* was followed by *Bacteroidota*, comprising 25.6 and 19.8% of the relative abundance, for HHS and LHS farms, respectively, and *Proteobacteria* represented 8.6 and 4.3% of the relative abundance for HHS and LHS. The relative abundance of all the *phyla* present is shown in Figure 2. A more similar distribution can be observed among the environmental samples (wall and slat), indicating a distinct pattern compared to the slurry samples.

Two hundred and fifty-five genera were identified across all the samples (wall, slat, and slurry). Among them, 4 genera were detected in all the samples analysed (*Corynebacterium*, *Turicibacter*, *Lactobacillus* and *Terrisporobacter*), a total of 18 genera were exclusive for HHS farms (*Terrabacter*, *Chryseobacterium*, *Soonwooa*, *Asteroleplasma*, *Pseudoramibacter*, *Butyrivibrio*, *Intestinimonas*, *Oscillospira*, *Anaerotruncus*, *Caproiciproducens*, *Schwartzia*, *Veillonella*, *Candidatus saccharimonas*, *Acetobacter*, *Devosia*, *Shewanella*, *Vitreoscilla* and *Morganella*) and 30 were exclusive for LHS farms (*Gordonia*, *Gelidibacter*, *Sulfuricurvum*, *Desulfatiglans*, *Desulfatiferula*, *Elusimicrobium*, *Endomicrobium*, *Caryophanon*, *Solibacillus*, *Atopostipes*, *Anaerofustis*, *Epulopiscium*, *Papillibacter*, *Sporobacter*, *Dehalobacterium*, *Hydrogenedesnsaceae*, *Dojkabacteria*, *Moranbacteria*, *Parcubacteria*, *Paenochrobactrum*, *Paracoccus*, *Mitochondria*, *Sphingopyxis*, *Thauera*, *Wohlfahrtiimonas*, *Aestuariicella*, *Rickettsiella*, *Oceanobacter*, *Thiopseudomonas* and *Arenimonas*).

Moreover, 11 were exclusive for wall samples (*Barchybacterium*, *Terrabacter*, *Micrococcus*, *Soonwoa*, *Desemzia*, *Veillonella*, *Devosia*, *Shewanella*, *Morganella*, *Serratia* and *Vibrio*); between them, *Terrabacter*, *Soonwoa*, *Veillonella*, *Devosia*, *Shewanella* and *Morganella* were only observed in wall samples collected from HHS farms. On the other hand, 5 genera were exclusive for slat samples (*Timotella*, *Savagea*, *Alloiococcus*, *Fusobacterium* and *Wohlfahrtiimonas*), of which *Wohlfahrtiimonas* was only detected in HHS farms. Finally, 147 genera were exclusive for slurry samples (the most prevalent were *Treponema*, *Fastidiosipila*, *Arcobacter*, *Trichococcus*, *Candidatus nomurabacteria*, *Proteiniphilum*, *Tissierella*, *Cloacimonas*, *Acholeplasma* and *Sedimentibacter*), of which 9 and 25 were exclusive for HHS and LHS samples, respectively.

### 3.3 Sanitary status environmental microbiota modulation

A PLS-DA with ALR-transformed variables was employed to evaluate the impact of sanitary status (HHS vs. LHS) on the environmental microbiota across wall, slat, and slurry samples. The analysis identified the most relevant genera, i.e., those that reached the highest prediction performance in each type of sample.

For three types of environmental samples, the model revealed a dependency of the microbiome on the farms´ sanitary status, distinguishing between both states effectively (Procrustes correlation and prediction performance). Wall samples, for instance, exhibited a high Procrustes correlation of 0.92, identifying 14 out of 57 genera as relevant variables and achieving prediction performance rates of 99.3% for HHS and 95.3% for LHS (Figure 2A). The results showed that 25% of the genera enable discrimination between both sanitary statuses in the wall samples, as listed in Table 2.

**Table 2:** Results of the Bayesian statistical analysis conducted on the key genera identified through partial least-squares discriminant analysis (PLS-DA) in wall samples.

Genus	HPD95	meanDiff	P0
*Aerococcus*	[−2.37, −0.44]	1.38	99.43*
*Leuconostoc*	[1.47, 2.22]	−1.34	99.23*
*Weissella*	[−0.22, 2.17]	−1.01	95.56*
*Lactococcus*	[0.65, 2.39]	−1.27	98.84*
*Jeotgalibaca*	[−2.36, −0.46]	1.19	98.08*
*Streptococcus*	[−0.13, 2.22]	−0.99	95.20*
*Clostridium_sensu_stricto_1*	[−2.39, −0.87]	1.25	98.79*
*Facklamia*	[−2.41, −0.37]	1.35	99.35*
*Carnobacterium*	[−0.73, 1.94]	−0.29	67.35
*Lactobacillus*	[−1.07, 1.74]	−0.30	68.07
*Jeotgalicoccus*	[−2.38, −0.39]	1.29	98.98*
*Enhydrobacter*	[−1.25, 1.53]	−0.25	65.47
*Aeromonas*	[−0.92, 1.84]	−0.63	84.27
*Um fam Enterobacteriaceae*	[−0.81, 1.88]	−0.70	87.18

HPD95 = The highest posterior interval density of 95% probability. P0 = Probability of the difference (DLHS-HHS) being greater than 0 when DLHS-HHS > 0 or lower than 0 when DLHS-HHS < 0. meanDiff = Posterior mean of the differences among the HHS and LHS samples. * = Significant differences were considered relevant if | DLHS-HHS | surpassed *R* value and its P0 > 0.95.

**Figure 3:**
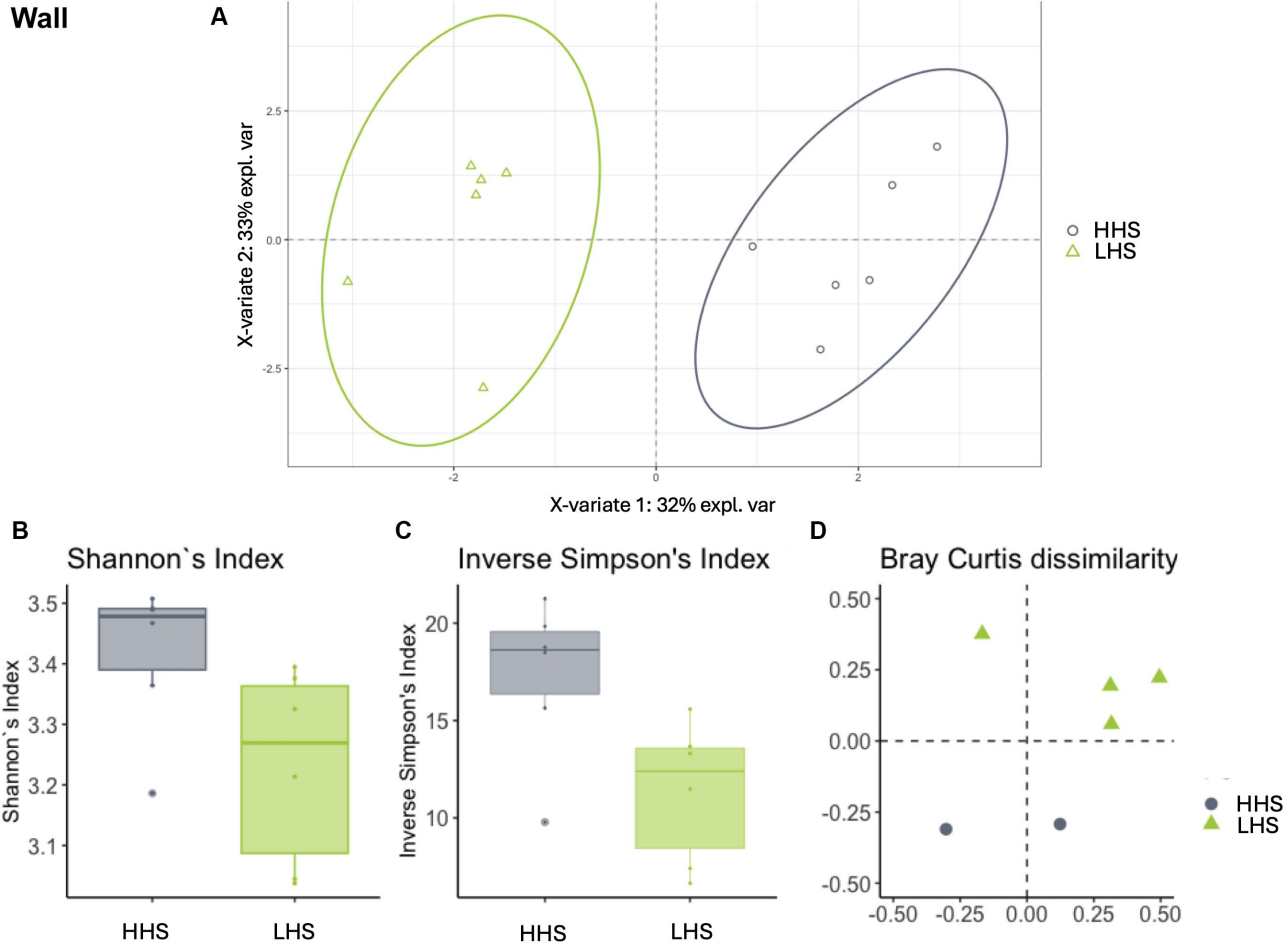
Environmental microbiota modulation induced by sanitary status in pig farms in slat samples. **(A)** Two-dimensional PLS-DA score plot was constructed using microbiota genera as variables, representing the distribution of the samples between the first two components in the model, which correspond to the sanitary status (HHS vs. LHS). Alpha diversity was computed using the **(B)** Shannon diversity index and **(C)** inverse Simpson index. Beta diversity was computed by calculating **(D)** the Bray–Curtis dissimilarity matrix. Differences among populations were established as having a *p*-value lower than 0.05. HHS, high health status; LHS, low health status.

Similarly, slat samples displayed a Procrustes correlation of 0.92, with 24 out of 95 genera identified as relevant variables, achieving a prediction performance rate of 93.0% for HHS and 100% for LHS (Figure 3A). Again, 25% of the genera were required for discriminating between the sanitary statuses in the slat samples, as detailed in Table 3.

**Table 3:** Results of the Bayesian statistical analysis conducted on the key genera identified through partial least-squares discriminant analysis (PLS-DA) in slat samples.

Genus	HPD95	meanDiff	P0
*Weissella*	[−2.28, 0.02]	−1.11	97.28*
*EscherichiaShigella*	[−1.72, 1.01]	−0.38	71.98
*Staphylococcus*	[−1.98, 0.64]	−0.72	87.80
*Clostridium_sensu_stricto_1*	[1.27, 2.31]	1.78	100*
*Terrisporobacter*	[0.9, 2.37]	1.64	99.97*
*Leuconostoc*	[−2.31, −0.21]	−1.27	98.90*
*Romboutsia*	[0.76, 2.34]	1.58	99.92*
*Macrococcus*	[−2.24, 0.08]	−1.05	96.32*
*Turicibacter*	[0.45, 2.37]	1.42	99.60*
*Lactococcus*	[−2.2, 0.25]	−0.95	94.27*
*Facklamia*	[0.61, 2.37]	1.50	99.83*
*Corynebacterium*	[0.94, 2.35]	1.67	99.99*
*Streptococcus*	[−2.07, 0.42]	−0.88	92.51
*Jeotgalibaca*	[0.25, 2.31]	1.32	99.08*
*Caryophanon*	[−0.05, 2.25]	1.10	97.10*
*Enterococcus*	[−2.08, 0.55]	−0.73	88.20
*Jeotgalicoccus*	[0.94, 2.4]	1.64	99.95*
*Rothia*	[−2.05, 0.58]	−0.73	87.82
*Um phylum Firmicutes*	[−0.23, 2.18]	1.02	95.53*
*Aerosphaera*	[−0.11, 2.22]	1.07	96.45*
*Proteus*	[−2.07, 0.48]	−0.80	89.99
*Solibacillus*	[−0.2, 2.21]	0.98	95.14*
*Chishuiella*	[−1.62, 1.18]	−0.21	63.02
*Um fam Enterobacteriaceae*	[−1.76, 0.96]	−0.41	74.35

HPD95 = The highest posterior interval density of 95% probability. P0 = Probability of the difference (DLHS-HHS) being greater than 0 when DLHS-HHS > 0 or lower than 0 when DLHS-HHS < 0. meanDiff = Posterior mean of the differences among the HHS and LHS samples. * = Significant differences were considered relevant if | DLHS-HHS | surpassed *R* value and its P0 > 0.95.

**Figure 4:**
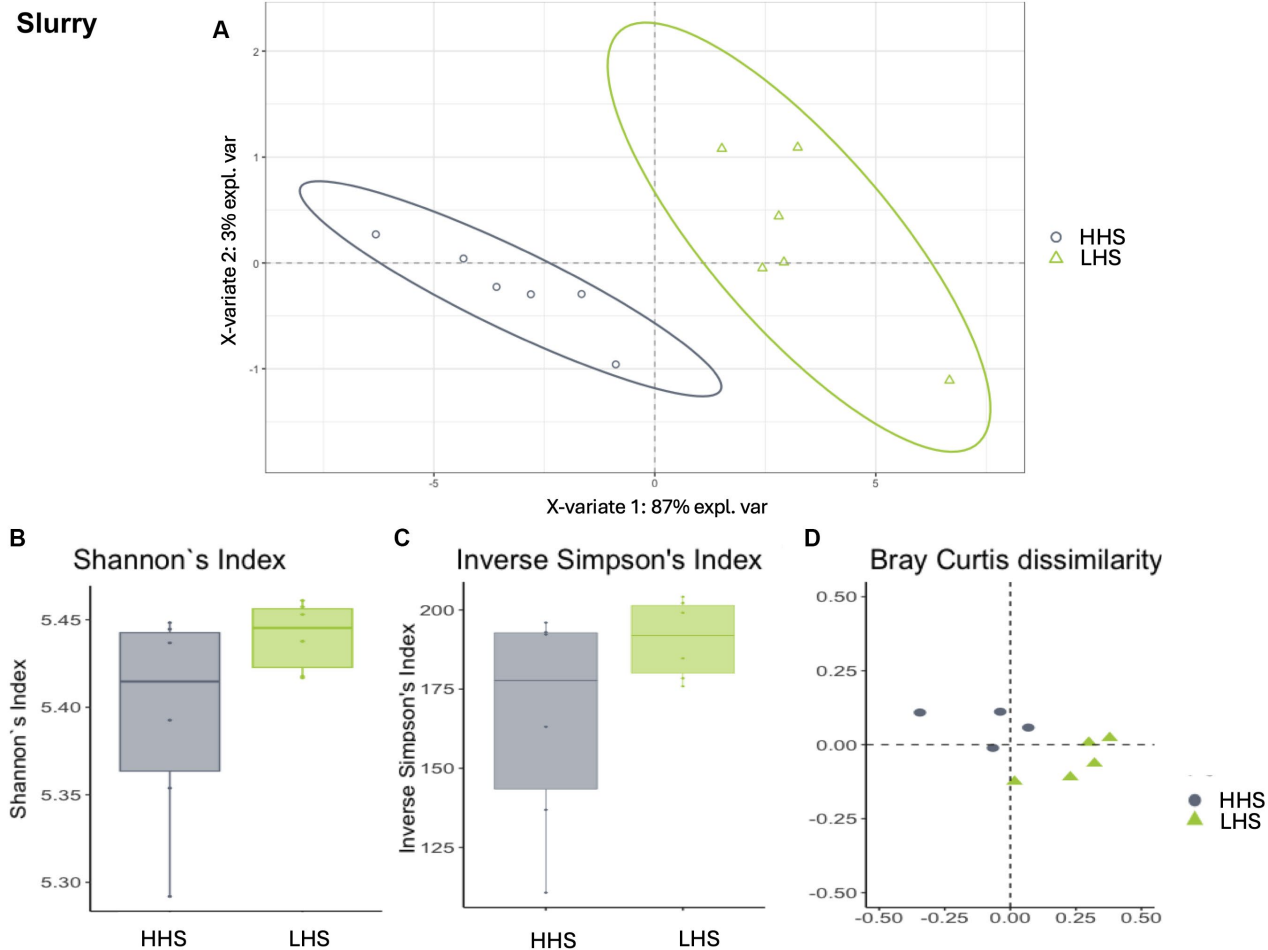
Environmental microbiota modulation induced by sanitary status in pig farms in slurry samples. **(A)** Two-dimensional PLS-DA score plot was constructed using microbiota genera as variables, representing the distribution of the samples between the first two components in the model, which correspond to the sanitary status (HHS vs. LHS). Alpha diversity was computed using the **(B)** Shannon diversity index and **(C)** inverse Simpson index. Beta diversity was computed by calculating **(D)** the Bray–Curtis dissimilarity matrix. Differences among populations were established as having a *p*-value lower than 0.05. HHS, high health status; LHS, low health status.

Finally, slurry samples also exhibited a high Procrustes correlation of 0.82, with 23 out of 259 genera identified as relevant variables, achieving prediction performance rates of 100% for HHS and 99.8% for LHS (Figure 4A). The results showed that only 8% of the genera enable discrimination between both sanitary statuses in the slat samples, as outlined in Table 4.

**Table 4:** Results of the Bayesian statistical analysis conducted on the key genera identified through partial least-squares discriminant analysis (PLS-DA) in slurry samples.

Genus	HPD95	meanDiff	P0
*Lactobacillus*	[−2.25, −1.35]	−1.81	100*
*DMER64*	[−1.13, 1.67]	0.27	66.11
*Um fam Planococcaceae*	[−2.41, −0.64]	−1.50	99.86*
*Bacteroidales_UCG001*	[−2.38, −0.82]	−1.60	99.94*
*Candidatus_Nomurabacteria*	[−1.65, 1.08]	−0.30	68.30
*Treponema*	[−2.39, −0.87]	−1.62	99.97*
*Fastidiosipila*	[−1.02, 1.71]	0.29	67.22
*Streptococcus*	[−2.31, −0.33]	−1.38	99.41*
*Um phylum Firmicutes*	[−2.31, −1.18]	−1.76	100
*Anaerocella*	[−2.37, −0.53]	−1.47	99.78*
*Actinomyces*	[−1.76, 1.01]	−0.37	71.78
*Um fam Synergistaceae*	[−1.78, 0.99]	−0.39	72.73
*Enterococcus*	[−2.38, −0.58]	−1.49	99.82*
*Proteiniphilum*	[−2.41, −0.53]	−1.46	99.74*
*Fibrobacter*	[−2.34, −1.1]	−1.72	99.99*
*Acidaminococcus*	[−2.41, −0.68]	−1.52	99.86*
*Jeotgalibaca*	[−2.39, −0.76]	−1.56	99.87*
*Um fam Acidaminococcaceae*	[−2.36, −0.69]	−1.55	99.87*
*Rikenellaceae_RC9_gut_group*	[−2.35, −1.08]	−1.72	100*
*Roseimarinus*	[−2.4, −0.54]	−1.46	99.76*
*Izemoplasmatales*	[−2.39, −0.58]	−1.50	99.79*
*NK4A214_group*	[−2.43, −0.78]	−1.57	99.92*
*Herbinix*	[−2.38, −0.68]	−1.52	99.86*

HPD95 = The highest posterior interval density of 95% probability. P0 = Probability of the difference (DLHS-HHS) being greater than 0 when DLHS-HHS > 0 or lower than 0 when DLHS-HHS < 0. meanDiff = Posterior mean of the differences among the HHS and LHS samples. * = Significant differences were considered relevant if | DLHS-HHS | surpassed *R* value and its P0 > 0.95.

Once the discriminative power of the model was established, the analysis of the microbiota composition was conducted, revealing similar patterns in both sanitary states for the environmental samples. The Shannon diversity index, which is more sensitive to species richness, indicated that microbiota diversity in wall, slat and slurry samples was similar under both sanitary conditions (Kruskal–Wallis test, HHS vs. LHS: *p*-value = 0.06, 0.06 and 0.13, respectively, Figures 2B, 3B, 4B). The inverse Simpson index, which is more sensitive to species evenness, revealed significantly higher diversity in HHS compared to LHS in wall samples (Kruskal-Wallis test, HHS vs. LHS: *p*-value = 0.03, Figure 2C), while no differences were observed in slat and slurry samples (Kruskal-Wallis test, HHS vs. LHS: *p*-value = 0.18, both, Figures 3C, 4C). Furthermore, pairwise PERMANOVA comparisons between groups using Bray–Curtis dissimilarity matrices showed similarity in microbiome composition between both sanitary statuses in wall, slat and slurry samples (*p*-value = 0.545, 0.058 and 0.324, respectively; Figures 2D, 3D, 4D).

To gain a deeper understanding of the impact of sanitary status on the environmental microbiome, a Bayesian statistical analysis was performed from the initial relevant genera identified through PLS-DA. For wall samples, as shown in Table 2, the Bayesian statistical analysis revealed 9 genera with relevant differences (P0 > 0.95) in abundance between the two sanitary statuses (HHS vs. LHS). Among them, *Leuconostoc, Weissella, Lactococcus*, and *Streptococcus* were more abundant in HHS; while *Aerococcus, Jeotgalibaca, Clostridium_sensu_stricto_1, Facklamia,* and *Jeotgalicoccus* were more abundant in LHS.

For slat samples, as shown in Table 3, the Bayesian statistical analysis revealed 9 genera with relevant differences (P0 > 0.95) in abundance between the two sanitary statuses (HHS vs. LHS). Among them, *Weissella, Leuconostoc, and Macrococcus* were more abundant in HHS; while *Clostridium_sensu_stricto_1, Terrisporobacter, Romboutsia, Turicibacter, Facklamia, Corynebacterium, Jeotgalibaca, Caryophanon, Jeotgalicoccus, UmphylumFirmicutes, Aerosphaera,* and *Solibacillus* were more abundant in LHS.

Finally, for slurry, as shown in Table 4, the Bayesian statistical analysis revealed 9 genera with relevant differences (P0 > 0.95) in abundance between the two sanitary statuses (HHS vs. LHS). Among them, all were more abundant in HHS: *Lactobacillus, UMFAMPlanococcaceae, Bacteroidales_UCG001, Treponema, Streptococcus, UmphylumFirmicutes, Anaerocella, Enterococcus, Proteiniphilum, Fibrobacter, Acidaminococcus, Jeotgalibaca, UmfamAcidaminococcaceae, Rikenellaceae_RC9_gut_group, Roseimarinus, Izemoplasmatales, NK4A214_group,* and *Herbinix.*

### 3.4 Bacterial functional annotation and distribution

In a general analysis all the genera identified in this study were included. A total of 33 categories of microbial functions linked to the bacterial communities were detected, of which 1 and 11 were exclusive for samples collected from HHS and LHS farms, respectively. As shown in Table 5, for both groups the predominant microbial functions assigned were chemoheterotrophy, fermentation, aerobic chemoheterotrophy and aromatic compound degradation. Moreover, as mentioned above, iron respiration was exclusive to HHS, and functions related to thiosulfate respiration, methanol oxidation, methylotrophy, nitrate denitrification, nitrite denitrification and respiration, denitrification, nitrous oxide denitrification and intracellular parasites were exclusive to LHS farms.

**Table 5:** OTUs abundance per functional group for high and low health status farms.

Group	HHS (%)	LHS (%)
Chemoheterotrophy	44.72	44.22
Fermentation	34.61	30.30
Aerobic chemoheterotrophy	10.27	13.52
Aromatic compound degradation	6.50	9.65
Animal parasites or symbionts	1.63	0.37
Nitrate reduction	0.79	0.08
Human associated	0.26	0.11
Human gut	0.21	0.06
Mammal gut	0.21	0.06
Respiration of sulphur compounds	0.17	0.41
Sulphate respiration	0.16	0.40
Cellulolysis	0.13	0.06
Nitrate respiration	0.07	0.07
Nitrogen respiration	0.07	0.07
Human pathogens all	0.05	0.06
Sulphite respiration	0.03	0.20
Dark hydrogen oxidation	0.02	0.10
Reductive acetogenesis	0.02	0.09
Aromatic hydrocarbon degradation	0.02	0.03
Aliphatic non methane hydrocarbon degradation	0.02	0.03
Hydrocarbon degradation	0.02	0.03
Sulphur respiration	0.01	0.01
Iron respiration	0.01	0.00
Thiosulfate respiration	0.00	0.01
Methanol oxidation	0.00	0.01
Methylotrophy	0.00	0.01
Nitrate denitrification	0.00	0.01
Nitrite denitrification	0.00	0.01
Nitrous oxide denitrification	0.00	0.01
Denitrification	0.00	0.01
Nitrite respiration	0.00	0.02

HHS, high health status farms; LHS, low health status farms.

Regarding the functional annotation for each sample type, functions associated with the microbiota of wall samples were fermentation, chemoheterotrophy, aerobic chemoheterotrophy, nitrate respiration and reduction and nitrogen respiration, all of them included genera identified in both, HHS and LHS farms. Regarding slat samples, the more relevant functions observed were fermentation and chemoheterotrophy, both related with the genera *Fusobacterium*, present in both experimental groups. Moreover, the functional annotation of more-environment-related samples (wall and slat) was performed. Results showed a total of 9 functional groups, of which the following are noteworthy: human pathogens, human-associated and animal parasites (related to *Moraxella* genus, and present in HHS and LHS samples), and nitrate and nitrogen respiration (related to *Shewanella* genus, only present in HHS samples).

Finally, for slurry samples, 29 functional groups were identified, of which it is interesting to highlight those related only to the genera *Paracoccus* (methanol oxidation, methylotrophy, nitrate denitrification, nitrite denitrification, nitrous oxide denitrification, and denitrification), aromatic compound degradation (*Gordonia* and *Desulfatiglans*), and intracellular parasites (*Mitochondria*), only identified in LHS farms.

## 4 Discussion

It is clearly demonstrated that controlling pathogenic microorganisms without the administration of antibiotics is one of the main challenges in modern livestock farming (35). Furthermore, strict antibiotic control in pig farming, the prohibition zinc oxide or the ban on highly effective field-level disinfectants such as formaldehyde make it very difficult to control animal diseases. After the implementation of all these mandatory regulations, this study has highlighted a significant difference in microbial communities established between farms that consistently experience health issues and those that consistently have good health outcomes. By understanding the differences between these populations in depth, we can develop tailored and effective protocols for each specific issue.

The microbial diversity of the samples was analysed according to alpha and beta diversity indexes. Although there were some differences in alpha diversity according to sample type (wall, slat or slurry), for all samples collected, significantly higher levels in beta diversity were observed for LHS farms. These findings are in agreement with data presented by Bridier et al. (2), which revealed that strict C&D procedures in pig slaughterhouses reduced the diversity of the bacterial community (2). This aligns with other studies indicating a decrease in microbial diversity when exposed to disinfectants (2, 36–40).

Regarding *phyla* composition, *Firmicutes* represented the dominant *phylum* of the environmental community in all the samples collected, followed by *Proteobacteria* in wall and slat samples, and by *Bacteroidota* in slurry samples. According to previous studies related to intestinal microbiota in pigs, *Firmicutes* is the dominant *phylum* during all the production cycle, followed by *Proteobacteria* at birth, and by *Bacteroidota* (formerly known as *Bacteroides*) during the rest of the production cycle, similar to the slurry results (41, 42). Both *phyla* are related to overall health of the animal, *Firmicutes* is involved in maintaining energy balance in the body, and *Bacteroidota* is associated with butyrate production and T cell-mediated immune responses, limiting the gastrointestinal colonisation by pathogens. In contrast, increased levels of *Proteobacteria* are repeatedly associated to intestinal inflammatory disorders (42–44).

At the genus level, 4 genera were present in all the samples analysed: *Corynebacterium*, *Turicibacter*, *Lactobacillus* and *Terrisporobacter*. Within them, *Lactobacillus* is known as a common inhabitant of the mammalian gastrointestinal tract, related to intestinal and immune system regulation, correct development of the intestinal microbiota and induction of competitive exclusion of pathogens by both adhesion to absorption sites of the intestinal mucosa and competition for nutrients (42, 45). Moreover, *Turicibacter* and *Terrisporobacter*, both significantly more abundant in LHS farms, are related to ether extract and protein digestibility, respectively, but considered opportunistic pathogens (41, 46, 47). However, the *Corynebacterium* genus, also significantly more abundant in LHS farms, contains opportunistic human pathogens asymptomatically carried by pigs (48).

Among the genera exclusively associated with HHS farms, beneficial and detrimental bacteria were observed. Some of the bacteria are related to beneficial effects to the host, such as *Butyrivibrio*, which belongs to butyrate-producing bacteria, with demonstrated beneficial effects on animals (49); *Oscillospira,* considered as a candidate for new probiotic generation with a strong association with body weight and animal health (50); *Caproiciproducens*, able to inhibit pathogenic bacteria, enhance animal immunity, and promote animal growth due to its ability to produce caproic acid (51, 52); or *Devosia*, a genus reported to be able to effectively reduce the toxicity of deoxynivalenol in diets for growing-finishing pigs (53). Furthermore, other genera are related to environmental health, such as *Terrabacter*, initially isolated from soil mixed with pig hair from Spain (54) and considered of ecological importance for its involvement in biological phosphate removal from wastewater and bioremediation processes (55). However, other genera were considered detrimental microbes, such as *Asteroleplasma* and *Intestimonas*, opportunistic pathogens related to heat stress and gut inflammation in growing pigs (50), *Candidatus saccharimonas*, involved in chronic inflammation, or *Shewanella* and *Morganella*, considered unusual opportunistic pathogens (56, 57).

Regarding the specific genera observed in LHS farms, more that are considered detrimental bacteria were observed. Some of them related to the host, such as *Solibacillus*, negatively correlated with crypt depth (58); or *Sporobacter*, suggested to have a negative role in gut health and associated with *Salmonella* positive free-range pigs (59, 60). There were also pathogens associated with ectoparasites, such as *Wohlfahrtiimonas*, causing several diseases in humans related to traumatic skin lesions, myiasis, wound contamination and sepsis (61); or *Rickettsia*, an obligate intracellular pathogen that causes diseases in humans, as well as domestic and wild animals, through vector transmission (62). Moreover, some genera were environment related, including *Gelidibacter*, associated with decomposition of pig carcasses (63); and *Sulfuricurvum* and *Desulfatiferula*, sulphur-reducing genera (64). On the other hand, two beneficial bacteria were detected, *Anaerofustis*, positively correlated with crude fibre digestibility (65), and *Arenimonas*, considered a soil probiotic (66).

Moreover, there were genera present in both experimental groups, but with significant differences in their abundance. Of these, noteworthy are 4 genera significantly more abundant in HHS farms: *Leuconosctoc*, with probiotic potential and antimicrobial effect such as lactic acid-producing bacteria (67); *Weissella*, also considered a potential probiotic with antimicrobial effect by producing compounds such as bacteriocins (68); *Lactococcus*, negatively correlated with methicillin-resistant *Staphylococcus* in healthy piglets, and with protective action in post-weaning diarrhoea and other intestinal infections in piglets (69, 70) and, in contrast, *Streptococcus*, one of the most important pathogens of pigs, causing septicemia, meningitis, arthritis, and endocarditis, especially in post-weaning pigs (71). Regarding the genera significantly more abundant in environmental samples (wall and slat) from LHS farms, it should be highlighted *Clostridium_sensu_stricto_1*, *Terrisporobacter*, and *Turicibacter*, previously associated to heat stress in pigs and considered opportunistic pathogens (46).

In addition, it is important to highlight some genera identified in the functional annotation analysis (FAPROTAX) because of their significant role in some important metabolic functions. There were some genera more-environmentally-related, including *Fusobacterium* (related to fermentation and chemoheterotrophy in slat samples), a genus with strong negative correlations with body weight gain in piglets and associated with diarrhoea and enteritis processes (72, 73); and *Moraxella* (human pathogen present in wall and slat samples), associated with infrequent opportunistic infections in humans and pigs. Furthermore, *Moraxella* has been described as a potential source of colistin resistance genes in pig farms (*mcr*-like genes, including new variants observed on pig production in China, *mcr* − 1.35 and *mcr*-1.36), as well as other antibiotic groups such as ampicillin, penicillin, quinolones, macrolides and tetracyclines (74, 75). Regarding the genera observed in each experimental group, it is important to note the role of *Shewanella* in iron respiration, a genus isolated from HHS group (as reported above). This process attracts much social and scientific attention due to its contribution to environmental pollution control and remediation field, promoting degradation of contaminants, such as aromatic compounds (76, 77). It is interesting to mention that many disinfectants used at the farm contain aromatic compounds among their active ingredients, that might have facilitated the persistence of this genus in the house environment. Finally, *Paracoccus*, a genus isolated from LHS farms and related to different functions (methanol oxidation, methylotrophy, nitrate denitrification, nitrite denitrification, nitrous oxide denitrification and denitrification), has previously been isolated from different environments, exhibiting excellent environmental adaptability, with a wide pH range and high concentrations of ammonia (78). In fact, it is reported that these bacteria facilitate different denitrification processes and participate in methanol oxidation, a process related to the presence of organic matter and exclusively from LHS farms (79, 80). However, it is essential to consider that functional annotations based on the FAPROTAX database provide inferred functions relying on 16S rRNA fragments, which may not be as precise as a comprehensive shotgun metagenomic study. For that reason, further studies such as metabolomics are needed to verify the functions and roles of these specific pathways in the different health status pig farms.

All the differences observed between the microbial populations of HHS and LHS, as well as the study of the functionality of the most prominent populations, emphasise the importance of knowing the individual situation of each farm when developing control methodologies, such as C&D protocols. With more specific, eco-friendly, and safe C&D protocols targeted against pathogenic microorganisms, we can achieve a much more sustainable livestock farming, as well as safer products for consumers and competitiveness in the market. While our results provide an initial insight into the relationship between environmental microbiota and farm health status, it’s important to note that pooling of samples was necessary to ensure adequate DNA quantity and quality. Further research is required to deepen our understanding of the role of environmental microbiota in farms with varying health statuses.

## Data availability statement

The datasets generated and analysed are available at NCBI’s BioProject PRJNA1079579 and BioSample SAMN40044702.

## Author contributions

CM: Conceptualization, Data curation, Funding acquisition, Investigation, Methodology, Project administration, Resources, Supervision, Validation, Visualization, Writing – original draft, Writing – review & editing. LM-G: Conceptualization, Data curation, Investigation, Methodology, Project administration, Resources, Supervision, Validation, Visualization, Writing – original draft, Writing – review & editing. JR: Investigation, Writing – review & editing. M-PV: Investigation, Writing – review & editing. MP-G: Investigation, Writing – review & editing. SV: Investigation, Writing – review & editing. CT-M: Investigation, Writing – review & editing. AM-F: Investigation, Writing – review & editing. LL-R: Data curation, Formal analysis, Investigation, Methodology, Software, Visualization, Writing – review & editing. LM-D: Data curation, Formal analysis, Investigation, Methodology, Software, Visualization, Writing – original draft, Writing – review & editing.
